# Exploring the potential of polyethylene terephthalate in the design of antibacterial surfaces

**DOI:** 10.1007/s00430-020-00660-8

**Published:** 2020-02-09

**Authors:** Tugçe Çaykara, Maria G. Sande, Nuno Azoia, Ligia R. Rodrigues, Carla Joana Silva

**Affiliations:** 1 CENTI-Center for Nanotechnology and Smart Materials, Rua Fernando Mesquita 278, 4760-034 Vila Nova de Famalicão, Portugal; 2 CEB-Centre of Biological Engineering, Universidade do Minho, Campus de Gualtar, 4710-057 Braga, Portugal

**Keywords:** Polyethylene terephthalate, Bacteria adhesion, Antibacterial properties, Surface functionalisation, Grafting, Topographical modification, Coating, Water contact angle

## Abstract

Polyethylene terephthalate (PET) is one of the most used polymeric materials in the health care sector mainly due to its advantages that include biocompatibility, high uniformity, mechanical strength and resistance against chemicals and/or abrasion. However, avoiding bacterial contamination on PET is still an unsolved challenge and two main strategies are being explored to overcome this drawback: the anti-adhesive and biocidal modification of PET surface. While bacterial adhesion depends on several surface properties namely surface charge and energy, hydrophilicity and surface roughness, a biocidal effect can be obtained by antimicrobial compounds attached to the surface to inhibit the growth of bacteria (bacteriostatic) or kill bacteria (bactericidal). Therefore, it is well known that granting antibacterial properties to PET surface would be beneficial in the prevention of infectious diseases. Different modification methods have been reported for such purpose. This review addresses some of the strategies that have been attempted to prevent or reduce the bacterial contamination on PET surfaces, including functionalisation, grafting, topographical surface modification and coating. Those strategies, particularly the grafting method seems to be very promising for healthcare applications to prevent infectious diseases and the emergence of bacteria resistance.

## Introduction

Bacterial infections are a big health issue, responsible for high expenditure and death. Among those, nosocomial infections are one of the most life threatening, given that in hospitals and other healthcare facilities the risk of infection is tremendously high. According to the European Centre for Disease Prevention and Control, more than 4 million people suffer from a healthcare associated infection in Europe every year [1].

Most bacteria exist in the form of a biofilm, which are microbial aggregates of diverse species that rely on extracellular products, such as extracellular polymeric substances (EPSs) expressed from the bacteria and a solid material surface. The expression of EPSs renders the attachment irreversible to the solid surface and once the bacteria are settled, synthesis of the bacterial flagellum is inhibited and the bacteria multiply rapidly, resulting in the development of a mature biofilm. At this stage, the bacteria form a resistant barrier to antibiotics, providing a source for systemic chronic infections. Therefore, despite the abundance of antimicrobial drugs and other modern antibacterial agents, bacterial infections still remain a threat to humanity, highlighting the urgent need to develop alternative ways to cope with infectious diseases [1].

A significant source of infections are surfaces of both indwelling medical devices or common utilities such as sinks, toilets, door handles, clothes, curtains or computer keyboards [1]. One strategy to prevent infections is to improve the material properties by making them anti-adhesive and/or biocidal. By the analysis of the state of the art, some contradictory results have been observed, mostly due to the particular experimental conditions applied in the different studies and the joint influences of the different material properties, such as roughness and surface energy, rendering it difficult to extract the exact influence of a material property on bacterial infections [2].

**Fig. 1:**
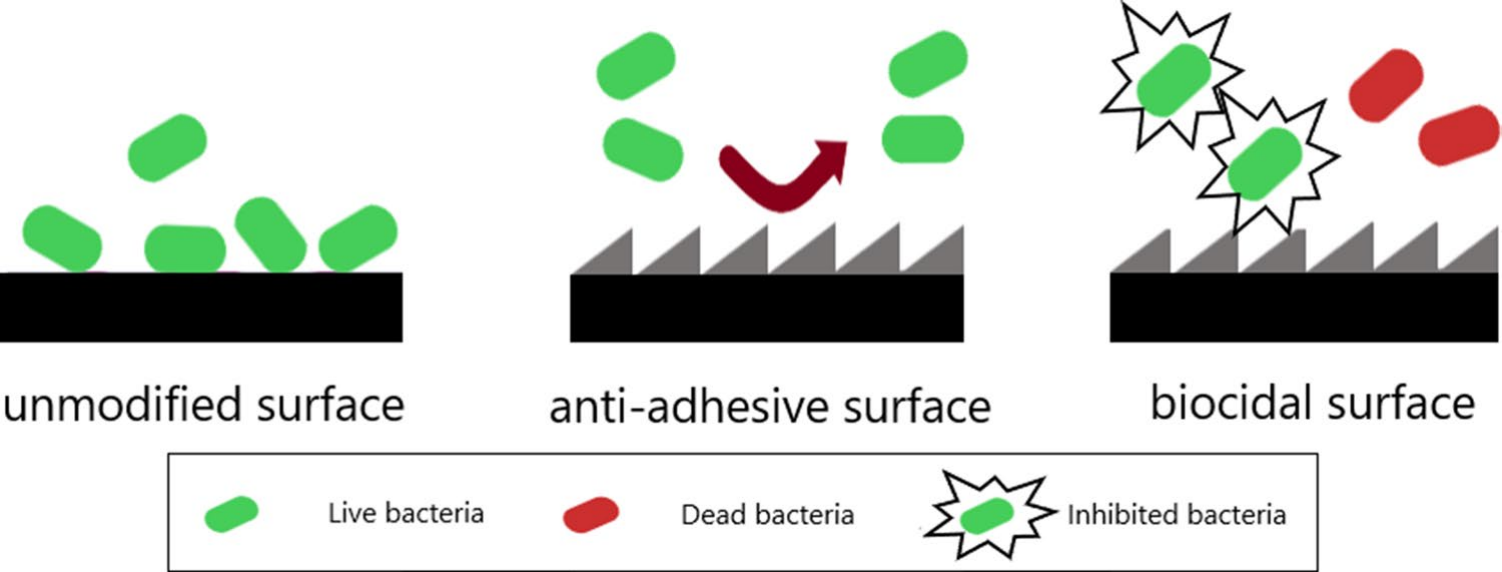
Surface action on anti-adhesive and biocidal surfaces

Material surface properties that have been implied in bacterial adhesion are surface charge and energy, hydrophilicity and surface roughness [2]. For instance, a negative surface generally exhibits a reduced bacterial adhesion due to the electric double layer repulsion, since most of bacterial cell surfaces carries a negative charge [3]. On the contrary, materials with positive charge can be used to inhibit bacterial growth, since they can attract and damage the bacterial cell walls, killing bacteria [4–9]. The increase in the surface free energy and the decrease in the contact angle leads to a reduced bacterial adhesion [10]. However, a superhydrophobic surface can also prevent bacterial adhesion due to the reduced protein adsorption and the entrapped air layer between the bacteria cells and the surface [2]. In addition, smooth surfaces exhibit less bacterial adhesion than rough ones, which present an increased area with favourable sites for bacteria to adhere [11]. Nevertheless, microtextured surfaces have shown less fouling compared to smooth surfaces in the cases where cells are slightly larger than the microtextured gaps. As such, there are a lot of different parameters that interfere with bacterial adhesion (and lately with biofilm formation), highlighting the need to properly modify and tune material surface properties to render them anti-adhesive, which is particularly useful for the health sector. Furthermore, the material surfaces can be modified with biocidal compounds like chitosan, nanoparticles, quaternary ammonium salts (QAS), triclosan and other antibiotics, to inhibit or kill bacteria [12, 13]. The simplified illustration of how surface properties can act on the reduction of bacterial contamination is shown in Fig. 1.

Currently, implants are made from a variety of synthetic fibres, being the majority made from polyethylene terephthalate (PET), expanded PET (ePET), polytetrafluoroethylene (PTFE), expanded polytetrafluoroethylene (ePTFE) and polyurethane (PU). However, PET, ePET and PTFE are the most commonly used fibres in commercial vascular prosthesis [14]. Other PET applications include sutures, heart valves, surgical meshes, scaffolds, urinary and bloodstream catheters. Its biocompatibility, high uniformity, mechanical strength and resistance against chemicals and/or abrasion make PET a promising material for several biomedical applications. Nevertheless, PET surfaces are prone to bacterial contamination and further modifications are necessary to limit and/or prevent such contamination [15]. This review will focus on the most recent strategies and methodologies being explored to confer PET surfaces a permanent antibacterial character.

## Surface modification methodologies

**Fig. 2:**
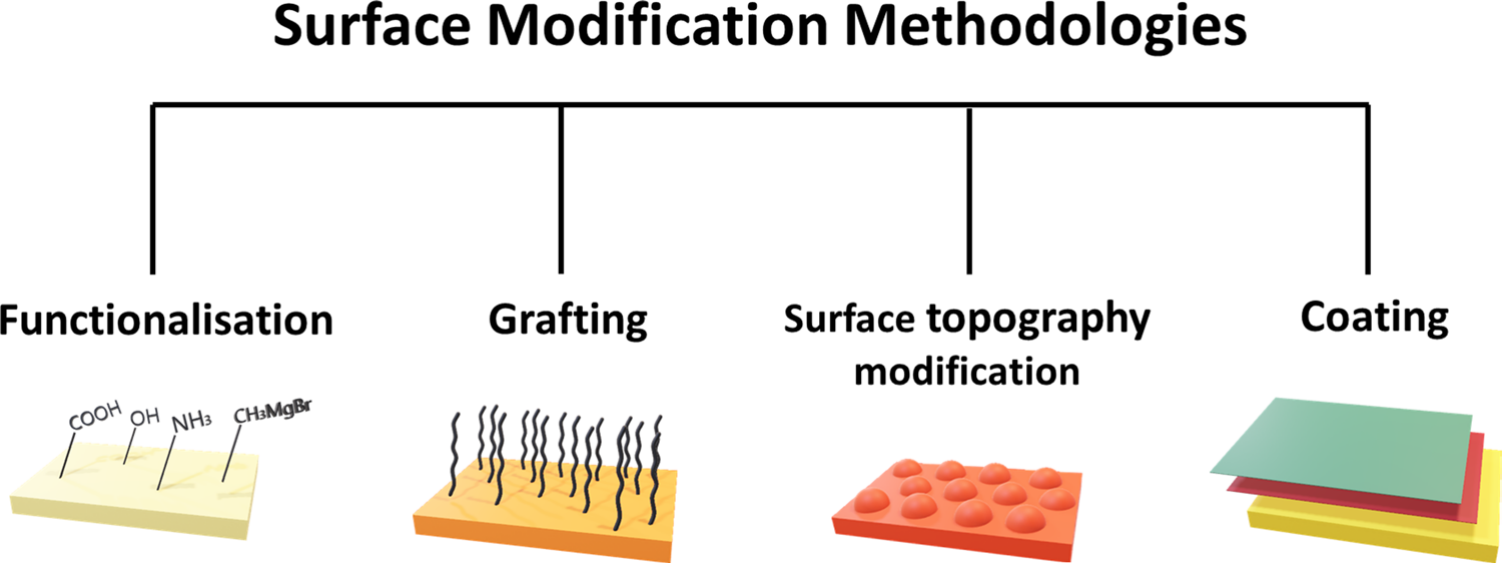
Surface modification methodologies used to develop PET antibacterial surfaces

The recent studies on antibacterial properties of PET have shown that several surface modification technologies have been used to limit and/or prevent bacterial contamination of PET, namely functionalisation, grafting, surface topography modification and coating. The methodologies are illustrated in Fig. 2.

While simple surface topography modification can provide anti-adhesive properties to plastic materials by changing surface properties like roughness, hydrophilicity and surface energy, both anti-adhesive and biocidal modifications can be obtained by the other depicted methodologies, depending on the technologies and chemicals used. Although significant antibacterial activity can be provided by grafting and coating methodologies, further studies needs to be performed to obtain PET surfaces that are able to completely avoid biofilm formation (and thus prevent bacterial contamination). When choosing a methodology for PET surface treatment, other two important factors need also to be considered: the durability of the superficial treatment and the cost–benefit ratio of the modified material. Table 1 presents a comparative overview of the surface modification technologies for PET, according to these three parameters. It should be noted that the methodologies vary within themselves and thus the generalised table might not give a straightforward comparison.

**Table 1:** Polyethylene terephthalate surface modification methodologies in relation to its antibacterial effectiveness, durability and cost

Methods	Antibacterial effectiveness	Durability	Cost–benefit
Functionalisation	*	**	**
Grafting	**	**	*
Coating	**	*	***
Surface topography modification	*	***	**

*Low degree; **medium degree; ***high degree

### Functionalisation

Several methods can be used for surface functionalisation of PET, including plasma treatment, ozone treatment, radiation, hydrolysis, oxidation and enzymatic modification, among others [15]. Plasma treatment, a commonly used method for surface functionalisation, is effective in creating hydrophilicity and increased roughness at the material surface which ultimately impact bacterial adhesion. Indeed, the surface of polymeric materials can be functionalised with different gas compositions and plasma conditions to enhance their hydrophilic or hydrophobic properties. While functionalisation with fluorine gas can lead to a more hydrophobic surface, functionalisation with air, oxygen, water vapour or carbon dioxide can improve polymer hydrophilicity [16, 17]. Junkar and co-workers [16] reported that oxygen plasma treatment of PET foils decreased the contact angle from 74° to 22° after a very short plasma treatment (3 s). For samples treated directly under the plasma glow, the water contact angle became extremely low (below 5°) after about 1 min of treatment. However, the authors also showed that the plasma treatment was not stable, since the obtained contact angle for the 3 s treated samples increased to 35° after 2 weeks of storage in air and remained constant after 4 weeks. In addition, the average roughness of those samples increased over 10 nm. It is important to notice that the selected functionalisation method can change the microstructure and nanostructure of a polymeric surface and this can change the surface roughness. The surface roughness by itself could also affect the hydrophilicity of the surface. Ahad and collaborators [18] studied the surface modification of PET films by irradiation with extreme ultraviolet photons to further evaluate the effects on the surface structure and wettability. Results showed that the water contact angle increased with an increased surface roughness. Roughness was found to be 6.6 nm and water contact angle was found to be 81.5° for unmodified PET samples, while the modified PET sample exhibited a maximum roughness value of 271 nm and a water contact angle of 98°.

Rezaei et al. [19] also showed that the increased surface energy accomplished by atmospheric plasma treatment can increase hydrophilicity on the material’s surface. The authors worked on the effects of different gases like helium, helium/oxygen and helium/nitrogen at different energy levels. It was shown that hydrophilicity, surface energy and surface roughness increased with an increased power. The lowest value for contact angle (25°) was obtained with the helium/nitrogen mixture, corresponding to the higher surface energy of 67 mN/m. The topographic analysis of the samples indicated a higher value of surface roughness for the samples treated with helium/oxygen plasma, showing that surface energy plays an important role on wettability. In addition, it has been shown that plasma treated samples led to a higher *Staphylococcus epidermidis* inhibition due to an increased surface free energy and decreased water contact angle. However, the material ageing altered the surface properties (hydrophilicity and surface energy, over time) that limited the bacterial inhibition [10].

Swar et al. [15] studied the potential use of Grignard reagents (methyl magnesium bromide (CH_3_MgBr) and dodecyl magnesium bromide (C_12_H_25_MgBr) to modify PET film and fabric surfaces. Modified PET surfaces showed decreased contact angles due to the exposed hydroxyl groups. The use of the methyl derivative Grignard reagent decreased water contact angle for both film and fabric samples, from 82° to 77° and from 111° to 102°, respectively. On the other hand, the water contact angle increased to 87° and 118° when the dodecyl magnesium bromide was used. This was due to the hydrophobicity of longer alkyl groups. The roughness of the film surface also increased from 6.69 to 18.05 nm when C_12_H_25_MgBr was used to modify the surface. The modified PET samples were found to be effective against *Staphylococcus aureus* and methicillin-resistant *S. aureus* (MRSA) growth, as well as against the formation of *Escherichia coli* biofilms.

Another example of PET functionalisation has been performed using benzophenone group terminated cationic quaternary ammonium salts (BP-QAS), which can interact with the cell membrane of bacteria and create reactive oxygen species (ROS) that can cause the death of bacteria. The modification of the PET samples has been done via photochemical hydrogen abstraction. Additional to bactericidal capacity, the modification of the surface has also enhanced hydrophilicity. The results showed that the modified surfaces presented more than 99% antibacterial efficiency against Gram-negative and Gram-positive bacteria [12].

### Grafting

**Fig. 3:**
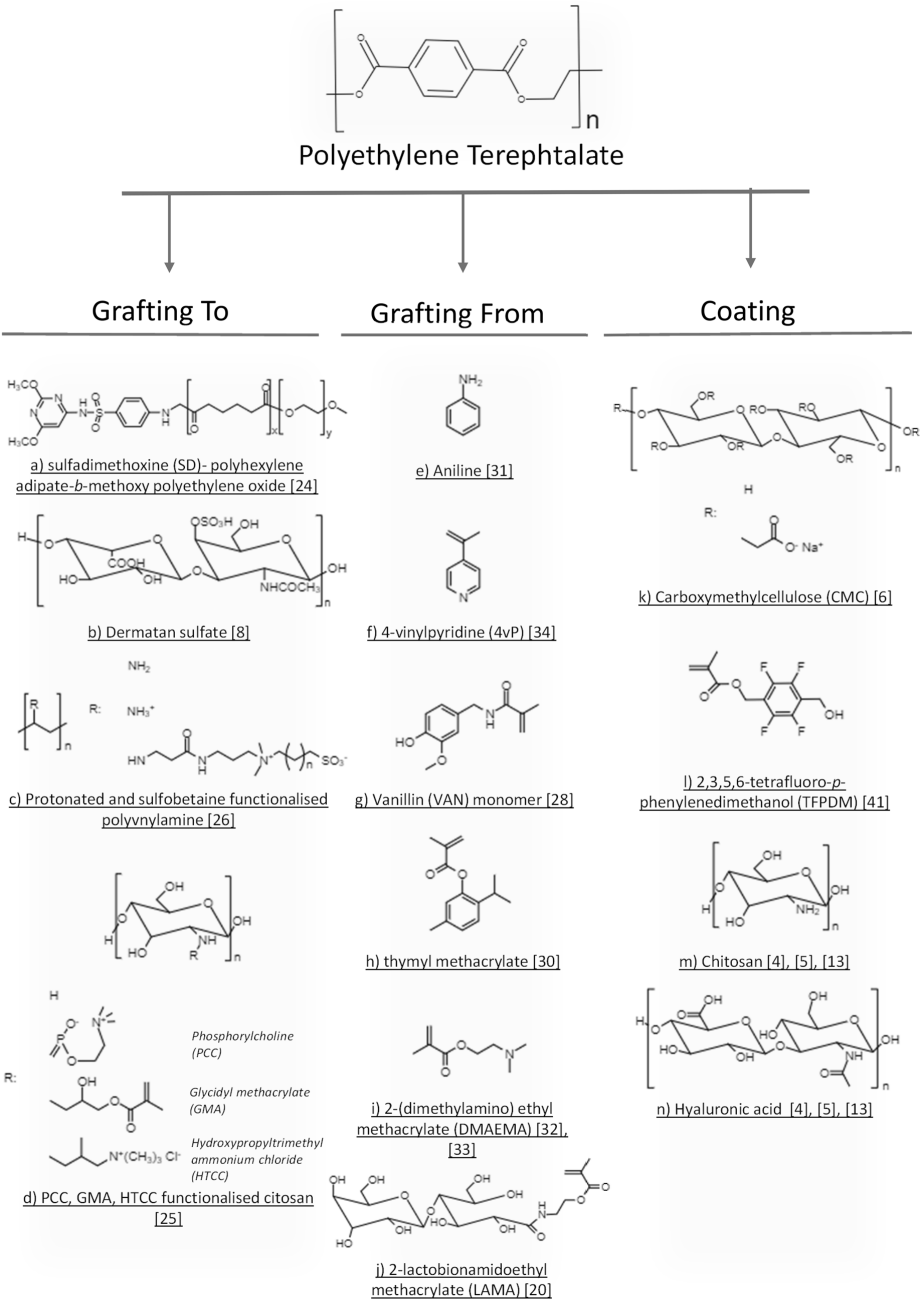
Chemical structure of some molecules used for surface modification of PET using “grafting to”, “grafting from” and coating methodologies

Numerous ways to develop an effective coating that has higher physical and chemical resistance to environmental conditions have been suggested [6, 7]; however, they are still limited by the type of the bonds created between the coating and the substrate. A grafting method, where covalent immobilization of the compounds takes place, is an alternative approach to create a resistant film at the polymer surface. The two main grafting methods being currently used are the “*grafting to*” and “*grafting from*”. While the “*grafting to*” method is used to attach polymer chains to the surface; in the “*grafting from*”, the monomer is attached to the surface and the polymerization occurs at the substrate’s surface [20]. Figure 3 shows the chemical structure of some molecules used for surface modification of PET using “*grafting to*”, “*grafting from*” and coating methodologies.

### “Grafting to” method

In the past years, there have been different attempts to prevent and/or inhibit bacterial contamination using the “*grafting to*” method. One of the interesting approaches was the immobilization of enzymes. Lysozyme is known for having bactericidal activity due to its capacity tohydrolyse the bacterial cell wall peptidoglycan (PG) and highly cationic conventional lysozyme types can kill the bacteria independently of cell wall PG damage [21]. Meslmani et al. [22] immobilized lysozyme onto woven and knitted crimped PET surfaces. Although the enzyme activity has been reduced to 55–60% with the grafting, the resulting samples were able to prevent bacterial adhesion. The anti-adhesive efficiencies of woven and knitted enzyme grafted PET were above 80% against *S. aureus* and *S. epidermidis*, and above 70% against *E. coli*, compared to unmodified PET samples.

The effectiveness of chitosan, a widely used natural polysaccharide, was investigated within the “*grafting to*” approach. Due to its positive charge, it has bacteriostatic and bactericidal effects by damaging the bacterial cell wall. Chitosan is broadly used in layer-by-layer methods to provide multifunctional films due to its partially positive charges. However, the layer-by-layer method does not provide stable coatings, because the layers are pH dependent and not resistant to abrasion [8]. Hayder et al. [8] covalently immobilized two separate layers of chitosan and dermatan sulfate (DS), an anionic polysaccharide, on PET using the coupling agent *N*,*N*′-dicyclohexylcarbodiimide (DCC). The results showed that chitosan and DS modification increased hydrophilicity. PET–DS–CHI was more hydrophilic exhibiting a contact angle of 71°, while PET–CHI–DS exhibited a contact angle of 87°. This was probably due to the higher amount of surface –COOH group on PET–DS–CHI. The tests against bacteria showed that both modified materials were more resistant to biofilm formation compared to the unmodified PET substrate, with PET–DS–CHI coating showing a better inhibition. However, PET–CHI showed a higher reduction of the bacterial adhesion which is thought to be due to the partial positive charges present on chitosan compared to other modified PET samples. The block copolymer of sulfadimehoxine polyhexylene adipate-*b*-methoxy polyethylene oxide (SD-PHA-*b*-MPEO) was another multifunctional copolymer studied and it is the combination of hydrophobic PHA to repel bacteria, hydrophilic MPEO to increase the host cell interactions for material integration in vivo and negatively charged SD which is a bacteriostatic antibiotic [23]. A porous structure has been obtained after its grafting onto PET by evaporating the solvent from polymer brush solution. Anti-adhesive efficiency of woven and knitted forms of modified PET samples were between 56–62% against *S. aureus* and *S. epidermidis*, while the efficiency was between 63–64% against *E. coli* [24].

Zwitterionic polymer brushes is an attractive approach for surface modification in the biomedical field, since it can provide good biocompatibility and anti-adhesive efficiency due to counteraction of the electrostatic hydration effect [25]. Timma and co-workers [26] developed polyvinylamine polymers that were functionalised with zwitterionic sulfobetaine side chains for PET fabrics. While sulfobetaine provided anti-adhesive properties, protonated amine groups provided bactericidal properties. A high substitution degree of sullfobetaine on the polymer chains might cause pure anti-adhesive properties. Therefore, reducing the substitution degree of sulfobetaine can allow a mixture of anti-adhesive and bactericidal properties due to the existence of uncoupled protonated amine groups. It was shown that the bacterial adhesion decreased with higher amount of substitution degree and 80% of substitution degree caused the material to lose almost all bactericidal properties. Moreover, the primary action of polymers with 60% of substitution degree seemed to be dependent of the fibre type. The bactericidal effect against Gram-negative bacteria was noted to be more influenced due to the different structure of the cell wall with the increasing substitution degree.

In another study done by Xv et al. [25], a zwitterionic glycidyl methacrylate–phosphorylcholine–chitosan (PCCs–GMA) was photo-immobilized on PET films. Hydroxypropyltrimethyl ammonium chloride chitosan–GMA (HTCC–GMA), cationic chitosan–GMA(Cs–GMA) and pristine PET were used to compare the results. The water contact angle was reduced to 34°, 36° and 47° after immobilization of PET–GMA–PCCs, PET–GMA–HTCC, PET–GMA–Cs, respectively. PET–GMA–PCCs has improved surface antibacterial properties and inhibited the adhesion up to 100% for *E. coli* and 92% for *S. aureus* compared with pristine PET. Although PET–GMA–HTCC and PET–GMA–Cs improved the antibacterial properties, many bacteria were observed on the surface of the tested materials. The live/dead bacteria assessment showed that no live or dead bacteria were observed on PET–GMA–PCCs surface, while there were some dead bacteria on PET–GMA–HTCC and some live bacteria on both pristine PET and PET–GMA–Cs. Both HTCC and chitosan are positively charged; however, the bacterial growth inhibition and contact killing properties of chitosan are limited in neutral conditions thus HTCC showed attached dead bacteria. Due to strong electrostatic hydration effect of zwitterionic PC, the attachment and killing of bacteria was supressed [25].

### “Grafting from” method

The “*grafting from*” is an alternative surface modification technology, being one of its main advantages the controllable molar mass and grafting density. Lepoittevin et al. [20] studied the grafting density by modulating the monomer/free initiator ratio. PET films were pre-treated with polyethylenimine (PEI), followed by the reaction with a surface initiator (bromoisobutyryl bromide). Finally, the Atom Transfer Radical Polymerization (ATRP) of 2-lactobionamidoethyl methacrylate (LAMA) was carried out and glycopolymer brushes were grown on the surface of the PET films with different grafting degrees. With the higher grafting degree, the authors obtained a water contact angle of 11° and a surface energy of 44.1 mN/m. The study showed the great potential for this type of carbohydrate and further studies should be performed to evaluate its potential for inhibiting bacterial adhesion.

Researchers have been showing an increased interest in the use of natural compounds as alternatives to synthetic active agents. Recent studies on some natural compounds like vanillin monomer which has bacteriostatic effect depending on target (more effective towards Gram-positive bacteria) [27] and thyme, which presents bacteriostatic and bactericidal effect, have been reported. In a study conducted by Mani and co-workers [28], the vanillin derived biobased monomer, *N*-(4-hydroxy-3-methoxybenzyl)-acrylamide (VAN), was used to modify the PET surface using the photopolymerization technique and *N*,*N*-diethylethylenediamine (DEDA) as a crosslinker. The surface modification with VAN reduced the contact angle from 80° to 62° and it was found that VAN grafted PET inhibited the adhesion of the Gram-positive bacteria *Rhodococcus wratislaviensis* and *S. aureus* by 85% and 97%, respectively. However, the inhibition of the Gram-negative bacteria *E. coli* and *Pseudomonas aeruginosa* was limited by 50%. *Rhizome Atractylodes macrocephala* (RAM), is another herbal product which has declared antibacterial properties. Shu et al. [29] worked with RAM grafting on PET non-woven substrates. They have found that the grafting of RAM was highly improved when pre-grafted polymerization of acrylic acid or plasma treatment was applied. It was further improved when both acrylic acid and plasma treatments were applied prior to grafting of RAM. The effectiveness against *S. aureus* and *E. coli* increased with the increased grafting percentage. Bedel et al. [30] performed ATRP polymerization of thymol monomer. The results showed that the water contact angle increased from 81° to 99° for samples treated with thymyl methacrylate. The total surface energy of PET was reduced from 44.7 to 40.5 mN/m. Furthermore, the thymyl methacrylate treated samples were highly antibacterial exhibiting up to a 99% decrease in the bacterial attachment against *P. aeruginosa*, *Listeria monocytongenes* and *S. aureus*.

In a recent study, Gallarato et al. [31] explored the antibacterial properties of polyaniline (PANI) coating grafted from PET. The authors further microstructured PANI coated surfaces with direct laser interface. They have found that the water contact angle increased from 72° to 84° for PANI coated PET film and it further increased to 101° with additional laser treatment on the PANI coating. PANI film reduced the bacterial adhesion of *P. aeruginosa* by 74%. Microstructure on PET–PANI film show to reduce the bacterial adhesion by 97%. The author also showed that the percentage of live bacteria was lower in modified surfaces and the live bacteria ratio was lower compared to dead bacteria on modified surfaces which proves its bactericidal effect.

A multifunctional coating where zwitterionic polymer brushes of polycarboxybetaine (PC) and polysulfobetaine (PS) formed on PDMAEMA (poly(2-(dimethylamino)ethyl methacrylate)) grafted PET sheets were developed by Jin and collaborators [32]. Both cationic killing behaviour from PDMAEMA and zwitterionic repelling behaviour from PS and PC have been obtained. Water contact angles of 30.4° and 30.6° with a polymerization time of 8 h have been obtained for PS and PC modified samples. A significant reduction of *E. coli* attachment to PC formed PDMAEMA grafted PET sheets was observed [33].

Further improvements in antibacterial properties can be also performed with additional functionalisation of grafted samples. Arslan et al. [34] studied the antibacterial effects of amine, chlorine, hydrogen peroxide, and triclosan functionalisation of grafted vinyl monomer on PET fibres. They have found that copolymerisation of vinyl monomers improved antibacterial properties and further functionalisation with triclosan showed the highest growth inhibition zone in all samples. The most promising vinyl grafting type with the bacterial inhibition was found to be the copolymerisation of 4-vinylpyridine which also gave higher inhibition zone compared to its oxidized or chlorine forms.

### Surface topography modification

As previously mentioned, surface roughness and topography greatly affect bacterial adhesion [2]. In addition, it has been shown that the surface roughness can affect the surface hydrophilicity. Surface wetting can either be homogeneous or heterogeneous, impacting differently the bacterial adhesion. The Wenzel’s phenomenon suggests that both hydrophilicity and hydrophobicity are enhanced by an increasing roughness on homogenously wetted surfaces, meaning that a hydrophilic surface will become more hydrophilic and a hydrophobic surface will become more hydrophobic [35]. The porous surfaces behave according to another phenomenon so-called the Cassie–Baxter phenomenon. In this state, the water droplet heterogeneously wet the surface and affect the wettability [36]. Gillett and collaborators [37] studied the effect of laser modification on surfaces, creating pit structures with 15 µm in diameter and 20 µm gap between each other on PET surfaces. The laser modification increased the roughness more than 30 times, from Ra = 0.81 µm to 30.1 µm. The surface modification has also affected the water contact angle, increasing it from 76.9° to 87.7°. Moreover, the modification was found to affect the *E. coli* distribution on the surface. Although more mature bacteria seem to accumulate around pits, there were no bacteria observed inside the pits. The authors suggested that this could be due to the presence of air pockets inside the pits following the Cassie–Baxter state. It should be noted that Gram-negative bacteria have an extra outer membrane which can ease the interaction with nano-irregularities [38].

Lithography is another method that can be used to modify the surface topography as an alternative to the laser surface modification. It has been reported that the size of the micropatterns can be arranged to prevent microorganisms to adhere and create biofilm onto the materials. Arisoy and co-workers [39] combined nanoimprinted shark skin pattern samples with 1.6 and 3 µm height, 1.3 and 2 µm width, 2.7 and 2 µm spacing by lithography with bactericidal effect of TiO_2_ on PET substrates. When shark skin patterned TiO_2_ samples were compared to smoother surfaces with the same chemistry, 70% reduction of the *E. coli* adhesion was observed. In addition, shark skin patterns led to 80% reduction in the bacterial adhesion as compared to flat PET surfaces. Moreover, it was shown that if the spacing is bigger than the width of the bacteria, then bacteria tend to adhere onto the surface between patterns rather than being repelled by them. Wang et al. [40] also evaluated the *E. coli* adhesion on micropatterned PET surfaces obtained by quartz photomask for six different pattern dimensions. The results showed that the shape of the microstructures affect the adhesion of cells and the live/dead cell ratio. In addition, the authors found the minimum adhesion with the smallest micropattern design (i.e., 1 µm).

### Coating

In the recent years, the layer-by-layer (LbL) methodology has been widely used. Within this method, the cationic and anionic polyelectrolyte layers can be bond through ionic bonds to form a thin coating film. Alvarez et al. [4] showed that the surface of a PET film could be successfully coated by positively charged chitosan and negatively charged hyaluronic acid to create a potentially antifouling surface. Chitosan has contact killing properties, while hyaluronic acid is hydrophilic, and it can repel bacteria due to a steric effect formed by water absorption. This type of coatings can have a nanometre scale thickness. Gallego et al. [5] used a similar method to coat the PET surface with chitosan and hyaluronic acid, obtaining coating thicknesses ranging from 45 to 385 nm depending on the number of bilayers (from 5 to 10). The water contact angle of the PET film was 77° and decreased to 54° after the deposition of the first bilayer. However, the contact angle has not shown any specific trend and varied between 54° and 77° with the further deposition of bilayers. The authors also observed a reduction of bacterial adhesion against *E. coli* with almost complete bacterial inhibition for ten layers of HA/CHI. However, it is important to bear in mind that the coating degradation is a problem when using the layer-by-layer surface modification. Indeed, the authors reported that 50% of the coating was degraded in the first 24 h and 90% during the first 6 days when exposed to enzymes. This value was 18% when enzymes were absent and remained stable for more than a month. Further incorporation of triclosan (TRI) and rifampicin (RIF) antibiotics into HA/CHI layer has been done and further antibacterial improvements have been observed. While the reduction in bacterial adhesion was found to be 80% for 5 bilayers of HA/CHI layers, it was found to be even higher > 99% for TRI and RIF incorporated HA/CHI layers [13]. An improvement of the material’s chemical and physical stability was attempted by Park et al. [6] by cross-linking the carboxymethylcellulose (CMC) polysaccharide and chitosan LbL-assembled multilayers on PETG (polyethylene terephthalate glycol modified) samples. The authors used a maximum of 20 bilayers of CMC/CHI to coat PETG and a thickness of 1818 nm was obtained, that was lower than 2 µ, which represents the thickness at which there is a risk of peeling. Moreover, the authors observed that when cross-linking was performed on 20 bilayers of CMC/CHI, the surface roughness increased from 20.3 to 57.7 nm. The cross-linking led to a super-hydrophilic surface exhibiting a reduction of the water contact angle from 35.34° to 4.86° (10 bilayers of CMC/CHI) and this change of the contact angle was thought to be due to surface roughness. Bacterial adhesion against *Streptococcus mutans* was reduced by 75% using cross-linked samples as compared to control PETG. Another study showed that an abrasion resistant coating incorporating chitosan can also be developed using a sandblasting method. Wieckiewiz et al. [7] formed a chitosan film layer on PET surfaces using the sandblasting method. Prior to chitosan coating, the silica coated sands formed a tribo-chemically hydrophilic adhesive silicate layer by high impact, to improve the stability.

In addition, fluoropolymers have been explored as antibacterial agents. Bao et al. [41] studied the use of 2,3,5,6-tetrafluoro-*p*-phenylenedimethanol (TFPDM) containing acrylate polymer blend (AF) and TFPDM sandwiched epoxy polymer structure (EF) to coat PET. While AF lowered the PET water contact angle from 60° to 41° and the roughness from 1.6 nm to 1.4 nm; EF lowered the water contact angle to 51° and the roughness to 1.3 nm. Both modified materials prevented the initial bacterial adhesion and biofilm formation by *Bacillus subtilis* and *E. coli*. However, AF performed slightly better reducing by 28% the *B. subtilis* adhesion and by 69% the biofilm formation, and by 89% the *E.* *coli* adhesion and by 94% its biofilm formation. It was also supported that the use of fluoride damages cell membrane by live/dead bacterial viability study.

The combination of topographical modification and coatings has been reported to increase its effectiveness. For instance, Yamada et al. [42] coated a PET film with nanoscale moth-eye cone-shaped protrusions from a hydrophilic resin made of urethane acrylate and polyethylene glycol (PEG) derivatives [43] with a size of approximately 200 nm in depth and diameter. Bacteria counts were reduced significantly with the use of moth-eye film compared to uncoated PET substrate due to specific structure of moth-eye film. It was also observed that the flat hydrophilic resin coated film reduced bacterial adhesion compared to uncoated PET film. This was attributed to bactericidal characteristics of PEG derivatives.

## Conclusion and outlook

In this review, recent surface modification approaches for granting antibacterial properties on PET were analysed according to the main methodologies used, namely, functionalisation, grafting, surface topography modification, coating and their combinations. Current developments show that treated PET surface presents a significantly higher antibacterial activity than the pristine form. The most popular methodologies for imparting antibacterial activity to PET surface appear to be the grafting and coating methods. High durability of the surface modification and preservation of PET mechanical properties are particularly important properties for in vivo applications, where material experiences excessive wear and thus must always be considered when modifying PET surface.

The studies also showed that coating and grafting methods can be advantageous for providing significantly higher hydrophilic surfaces, being the water contact angle one of the most used characterization methods. Low contact angles were found in literature (around 4° with the coating method and around 11° with the “*grafting from*” method) for treated polyethylene terephthalate. As stated by several authors, a surface with a high hydrophilicity behaviour (low contact angle) shows a great potential for developing anti-adhesive PET products for the medical sector. Further topographical surface modifications after grafting methods have been also used to improve the materials antibacterial properties.

Furthermore, combining anti-adhesive properties with bactericidal properties is a popular strategy for achieving high antibacterial efficiencies for PET materials. However, one must consider the potential bacterial resistance, the cost–benefit ratio, the durability of the modification, as well as the possible toxic effects of the antibacterial agents on the environment when choosing the most suitable modification approach for the envisaged PET products. Moreover, it is also important to keep in mind that different types of bacteria react differently to the presence of bactericidal agents due to their different shape (spheres, rods or spirals) and outer membrane structure.

Finally, the use of natural and biological molecules to improve the antibacterial properties of PET materials is a very promising approach to further develop new strategies against infectious diseases.
